# Comparison of microscopic and xpert MTB diagnoses of presumptive mycobacteria tuberculosis infection: retrospective analysis of routine diagnosis at Cape Coast Teaching Hospital

**DOI:** 10.1186/s12879-024-09566-9

**Published:** 2024-07-02

**Authors:** Kwame Kumi Asare, Daniel Edem Azumah, Czarina Owusua Adu-Gyamfi, Yeboah Kwaku Opoku, Edward Morkporkpor Adela, Philip Afful, Godwin Kwami Abotsi, Ernest Awuakye Abban, Paul Ekow Duntu, Akwasi Anyamful, Alberta Bedford Moses, Emmanuel Botchway, Philimon Mwintige, Samuel Kyei, Linda Eva Amoah, Emmanuel Owusu Ekuman

**Affiliations:** 1 Biomedical and Clinical Research Centre, College of Allied Health Sciences, University of Cape Coast, Cape Coast, Ghana; 2 Laboratory Departments, Cape Coast Teaching Hospital, Cape Coast, Ghana; 3 Department of Biology Education, Faculty of Science Education, University of Education, Winneba, Ghana; 4 Department of Biomedical Sciences, School of Allied Health Sciences, College of Allied Health Sciences, University of Cape Coast, Cape Coast, Ghana; 5 Department of Medical Laboratory Science, School of Allied Health Sciences, College of Allied Health Sciences, University of Cape Coast, Cape Coast, Ghana; 6 Department of Medical Biochemistry, School of Medical Sciences, College of Health and Allied Sciences, University of Cape Coast, Cape Coast, Ghana; 7 Department of Optometry and Vision Science, University of Cape Coast, Cape Coast, Ghana; 8 Department of Immunology, Noguchi Memorial Institute for Medical Research, University of Ghana, Accra, Ghana

**Keywords:** *Mycobacterium tuberculosis* (MTB*)*, TB diagnosis, GeneXpert MTB/RIF, Cape Coast Teaching Hospital (CCTH), Ghana

## Abstract

**Introduction:**

*Tuberculosis* is a global health problem that causes 1. 4 million deaths every year. It has been estimated that sputum smear-negative diagnosis but culture-positive pulmonary TB diagnosis contribute to 12.6% of pulmonary TB transmission. TB diagnosis by smear microscopy smear has a minimum detection limit (LOD) of 5,000 to 10,000 bacilli per milliliter (CFU/ml) of sputum result in missed cases and false positives. However, GeneXpert technology, with a LOD of 131–250 CFU/ml in sputum samples and its implementation is believe to facilitate early detection TB and drug-resistant TB case. Since 2013, Ghana health Service (GHS) introduce GeneXpert MTB/RIF diagnostic in all regional hospitals in Ghana, however no assessment of performance between microscopy and GeneXpert TB diagnosis cross the health facilities has been reported. The study compared the results of routine diagnoses of TB by microscopy and Xpert MTB from 2016 to 2020 at the Cape Coast Teaching Hospital (CCTH).

**Methods:**

The study compared routine microscopic and GeneXpert TB diagnosis results at the Cape Coast Teaching Hospital (CCTH) from 2016 to 2020 retrospectively. Briefly, sputum specimens were collected into 20 mL sterile screw-capped containers for each case of suspected TB infection and processed within 24 h. The samples were decontaminated using the NALC-NaOH method with the final NaOH concentration of 1%. The supernatants were discarded after the centrifuge and the remaining pellets dissolved in 1–1.5 ml of phosphate buffer saline (PBS) and used for diagnosis. A fixed smears were Ziehl-Neelsen acid-fast stain and observed under microscope and the remainings were used for GeneXpert MTB/RIF diagnosis. The data were analyze using GraphPad Prism.

**Results:**

50.11% (48.48–51.38%) were females with an odd ratio (95% CI) of 1.004 (0.944–1.069) more likely to report to the TB clinic for suspected TB diagnosis. The smear-positive cases for the first sputum were 6.6% (5.98–7.25%), and the second sputum was 6.07% (5.45–6.73%). The Xpert MTB-RIF diagnosis detected 2.93% (10/341) (1.42–5.33%) in the first and 5.44% (16/294) (3.14–8.69%) in the second smear-negative TB samples. The prevalence of Xpert MTB-RIF across smear positive showed that males had 56.87% (178/313) and 56.15% (137/244) and females had 43.13% (135/313) and 43.85% (107/244) for the first and second sputum. Also, false negative smears were 0.18% (10/5607) for smear 1 and 0.31% (16/5126) for smear 2.

**Conclusion:**

In conclusion, the study highlights the higher sensitivity of the GeneXpert assay compared to traditional smear microscopy for detecting MTB. The GeneXpert assay identified 10 and 16 positive MTB from smear 1 and smear 2 samples which were microscopic negative.

## Introduction

*Mycobacterium tuberculosis* (MTB) is estimated to infect 8.7 million new people and causes approximately 1.4 million deaths yearly [1, 2]. The rate of MTB infections and death is alarming and remains a public health problem globally [3]. The rapid diagnosis of MTB is a challenge, especially among low and middle-income countries, where over 90% of TB cases reside [4, 5]. In addition, there is the extensive development of drug resistance in the ongoing transmission of TB disease [6].

TB diagnosis in developing countries is mainly based on smear microscopy for acid-fast bacillus or by a culture, and in some clinical scenarios and chest X-ray findings [7, 8]. Although Culture method remains the gold standard for TB diagnoses, the method requires cumbersome laboratory procedures and sophisticated biological safety equipment that are very expensive to operate in resource-limited countries [9, 10]. Sputum smear microscopy is the cheapest TB diagnostic tool but has the lowest sensitivity [11]. There are technical challenges in TB slide preparation including generation of droplet nuclei, inhalation risks, and transmissibility [12, 13].

The transmissibility of missed TB cases from sputum smear-negative diagnosis resulting from lower sensitivity is a global health concern [14, 15]. Previous studies have estimated that sputum smear-negative diagnosis but culture-positive pulmonary TB diagnosis contribute to about 12.6% of pulmonary TB transmission [16–18]. This suggests that more sensitive and specific diagnostic tools with rapid turnaround time are required to provide timely diagnosis and prevent delayed case detection, suffering, death, and disease transmission. Prompt and efficient diagnosis is essential for TB control, prevention, and eradication and also provides a timely intervention to prevent TB drug resistance, especially the frontline anti-TB drugs isoniazid (isonicotinic acid hydrazine), pyrazinamide, ethambutol and rifampicin (RIF).

GeneXpert MTB/RIF assay is a molecular-based diagnostic tool that detects MTB and RIF resistance [10, 14, 18]. The World Health Organization (WHO) has recommended the Xpert MTB/RIF diagnostic tool for national tuberculosis programs in developing countries due to its high sensitivity and specificity for TB detection, easy use, automation, and very rapid turnaround time of 2 h [19, 20]. The Xpert MTB/RIF diagnosis operates based on nested real-time PCR [21, 22]. The Xpert MTB/RIF also has the advantage of requiring minimal biosafety facilities, and it is not prone to cross-contamination and is very efficient for TB diagnosis [23–25].

Microscopy-based tuberculosis (TB) diagnosis offers various obstacles, including low sensitivity and specificity, with a minimum detection limit (LOD) of 5,000 to 10,000 bacilli per milliliter (CFU/ml) of sputum, which can result in missed cases and false positives [26]. Accurate results necessitate qualified technicians and high-quality samples, and maintaining the necessary laboratory equipment and supplies can be challenging in resource-constrained environments [27]. In early 2013, light-emitting diode (LED) microscopes were installed at 156 high-burden sites to minimize workload and speed up TB detection. Additionally, GeneXpert technology, with a LOD of 131–250 CFU/ml in sputum samples, was implemented in selected locations to improve TB diagnosis, particularly among people living with HIV (PLHIV) and children, and to facilitate the early detection of drug-resistant TB by the Ghana Health Service (GHS) [28]. This project has been extremely successful, resulting in the installation of additional machines in all Regional Hospitals and Teaching Hospitals across the country. Currently, 134 GeneXpert machines have been bought and disseminated around the country to improve TB diagnosis among PLHIV and ensure early detection of drug-resistant cases. However, no study has been conducted to examine the pattern of TB diagnosis between microscopy and GeneXpert. The study compared the results of routine diagnoses of TB by microscopy and Xpert MTB from 2016 to 2020 at the Cape Coast Teaching Hospital (CCTH).

## Materials and methods

### Study design and area

The study compared routine TB diagnosis results at the Cape Coast Teaching Hospital (CCTH) from 2016 to 2020 retrospectively. The Xpert MTB-RIF method was compared to the microscopy TB smear method from 2016 to 2020 at CCTH in the Central Region of Ghana. The Central Region of Ghana spans an area of 9,826 square kilometers, comprising about 6.6% of the country’s total land area. It is one of Ghana’s sixteen administrative regions, bordered by the Gulf of Guinea to the south, the Western and Western North Regions to the west, the Greater Accra Region to the east, the Ashanti Region to the north, and the Eastern Region to the northeast. The region consists of 22 administrative districts, with Cape Coast as the capital. Approximately 63% of the population lived in rural areas as of 2008. Predominantly inhabited by the Akan people, the majority are Fantes. As of 2020, the estimated population was 2,605,490, with a regional growth rate of 3.1% and a population density of about 215 inhabitants per square kilometer. The CCTH is the only teaching hospital in the Region and serves as the referral hospital for the Region. In all, 6019 suspected TB cases visited the Microbiology Laboratory of CCTH between 2016 and 2020 for TB diagnosis. Of the 6019 suspected TB, 2393 (39.8%) cases were referrals from various health facilities across the Region.

### Ethical approval

The Biomedical and Clinical Research Centre Research Board, University of Cape Coast, approved the study (BCRC/22/03_0001/01). CCTH permitted the analyses of routine TB data from the hospital. Since this study used retrospective data, consent to participate and or for publication did not apply. All data obtained from laboratory records were anonymous.

### Mycobacteria Tuberculosis (TB) diagnostic strategy at CCTH

**Fig. 1:**
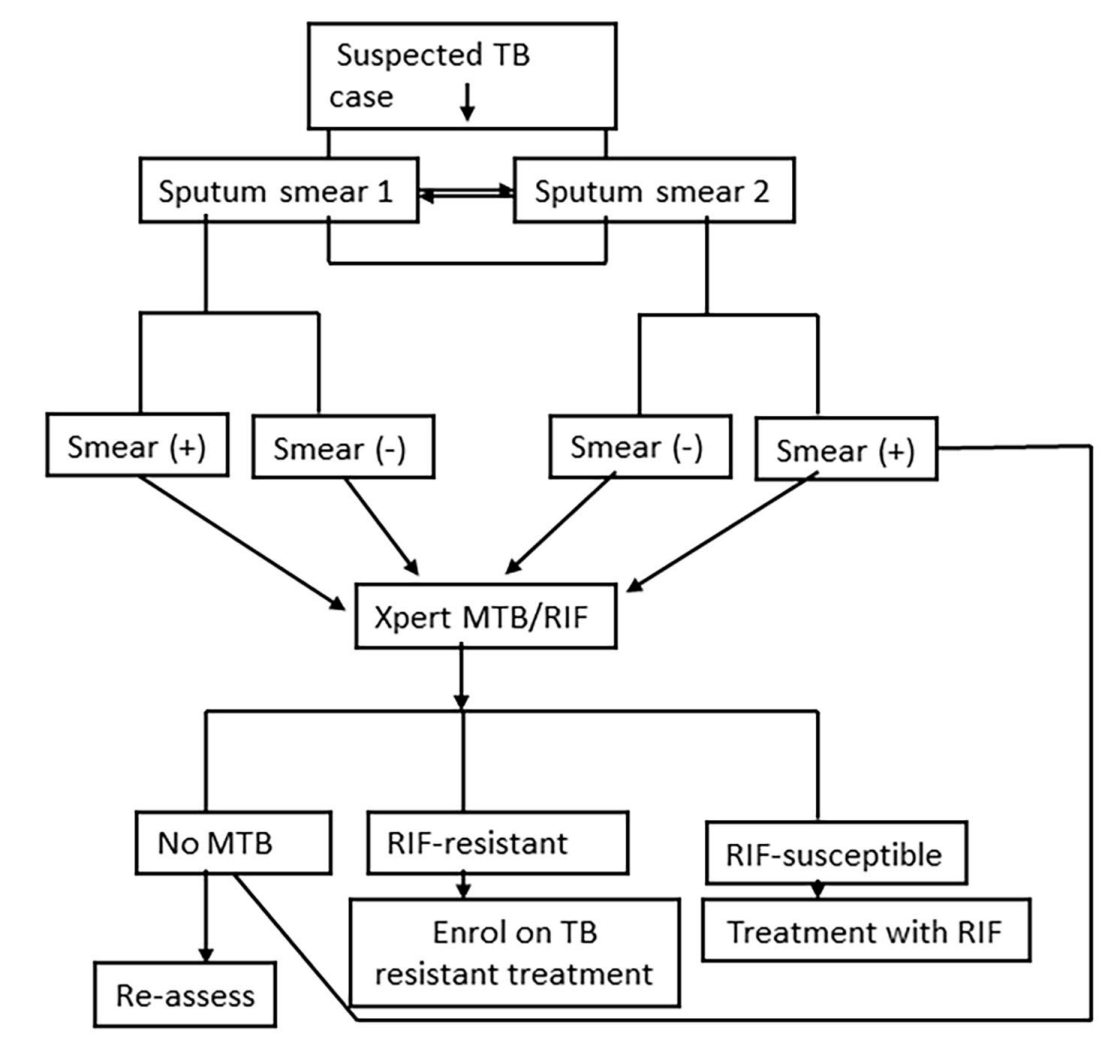
Diagnostic strategy of Mycobacteria tuberculosis (MTB)

Two sputum samples were collected from each presumptive TB individual at an interval of three or more hours. The TB smears were prepared from each sputum sample for microscopic diagnosis (Smears from the initial sputum sample were labeled as TB smears 1 and the smear from the second sputum sample was labeled as TB smear 2). Similarly, the Xpert MTB-RIF diagnosis was conducted for all the collected sputum samples. The second sputum was collected three hours or more after the collection of the first sputum sample to increase the chance of capturing the shed TB in the sputum and also to reduce the turnaround time for TB diagnosis and treatment. The Xpert MTB-RIF test was performed for smear-positive (+) and smear-negative (-) TB cases (Fig. 1).

### Sample collection

Sputum specimens were collected into 20 mL sterile screw-capped containers for each case of suspected TB infection and processed within 24 h by a laboratory technologist as recommended by the World Health Organisation WHO [29].

### Decontamination of the sputum samples

The samples were decontaminated using the NALC-NaOH method with the final NaOH concentration of 1% [30–32]. The supernatants were discarded after the centrifuge and the remaining pellets dissolved in 1–1.5 ml of phosphate buffer saline (PBS) and used for diagnosis.

### Microscopy

A fixed sputum smear (smears 1 and 2) was made using the pellets and stained with Ziehl-Neelsen acid-fast stain [33, 34]. Positive slides with acid-fast bacilli (AFB) were reported based on the following criteria at a 400X magnification: no AFB seen was considered a negative, 1 to 9 AFB per 100 fields was considered rare (scanty), 10–99 AFB per 100 fields was considered moderate (positive +), 1–10 AFB per field (check 50 fields) (positive ++), greater than 100 AFB per 100 fields was considered many (positive +++) according to Samuel & Kanna, 2022 [35].

### Xpert MTB/RIF Diagnosis

All sputum samples were processed and diagnosed using the Xpert MTB/RIF [25]. The test uses the G4 version of the cartridges. Decontaminated sputum samples were neutralized with an excess of 0.067 M phosphate buffer containing 0.2% phenol red, adjusting the pH to 6.8 ± 0.2 using 4% sodium hydroxide or 1 M hydrochloric acid as necessary. The neutralized samples were then centrifuged at 3000× g for 15 min, after which the supernatant was carefully discarded. The pellet obtained from centrifugation was used for further tuberculosis diagnostic testing. All the processes followed the manufacturer’s instructions (Cepheid, Sunnyvale, CA).

### Statistical analysis

The data that was entered into the Excel spreadsheet were cross-checked by two independent researchers to validate the data on Excel to the recordings in the Laboratory Logbooks and analyzed using Excel 2016 (Microsoft Corporation). The results of this study are presented in tables and figures. The data are in frequencies and percentages. The Clopper-Pearson test was used to determine the confidence intervals of proportions of relevant outcome variables under study. The odds ratios and associated 95% confidence intervals were used to assess the odds of stratified variables by sociodemographic and clinical variables. All data analyses were performed using GraphPad version 9.3.1.

## Results

### Characteristics of the presumptive TB cases

The CCTH TB clinic tested 6019 suspected TB cases from 2016 to 2020. A total 39.9% (95% CI; 38.67-41.16%) of the presumptive TB cases were referrals from other health facilities across the Central region of Ghana. Of the cases, 50.11% (48.48–51.38%) were females with an odd ratio (95% CI) of 1.004 (0.944–1.069) more likely to report to the TB clinic for presumptive TB diagnosis. Of the presumptive TB cases, 52.69% (51.41–53.97%), 23.98% (22.89–25.09%), and 17.8% (16.84–18.8%) were among the age categories 31–60 years, 16–30 years and greater than 60 years age categories respectively. The smear-positive cases for the first sputum were 6.6% (5.98–7.25%), and the second sputum was 6.07% (5.45–6.73%). The positive Xpert MTB-RIF diagnosis among smear-positive samples was 83.59% (79.56–87.1%) and 83.9% (79.58–87.7%) for the first and second sputum. Also, the smear-negative samples tested positive by the Xpert MTB-RIF diagnosis for the first and second sputum were 2.93% (1.42–5.33%) and 5.44% (3.14–8.69%) (Table 1).

**Table 1:** Demographic characteristics of the study subjects with suspected Mycobacteria Tuberculosis infections

Characteristics	*n*/*N* (%)	95% CI	OR (95% CI)
**Age categories/years**			
< 5 y	34/5914 (0.57)	0.4–0.8	0.006 (0.004–0.01)
5–15 y	29/5914 (0.49)	0.33–0.7	0.005 (0.003–0.01)
16–30 y	1418/5914 (23.98)	22.89–25.09	0.315 (0.294–0.34)
31–60 y	3116/5914 (52.69)	51.41–53.97	1.114 (1.046–1.19)
> 60 y	1053/5914 (17.80)	16.84–18.8	0.217 (0.201–0.23)
**Sex**			
Male	3003/6019 (49.89)	48.62–51.16	0.996 (0.936–1.06)
Female	3016/6019 (50.11)	48.84–51.38	1.004 (0.944–1.07)
**Health facility**			
CCTH	3603/5996 (60.09)	58.84–61.33	1.506 (1.414-1.60)
Referral	2393/5996 (39.91)	38.67–41.16	0.664 (0.624–0.71)
**Diagnosis**			
**Smear 1**			
Pos	396/6003 (6.60)	5.98–7.25	0.071 (0.063–0.08)
Neg	5607/6003 (93.40)	92.75–94.02	14.14 (12.7-15.75)
**Smear 2**			
Pos	331/5457 (6.07)	5.45–6.73	0.065 (0.06–0.07)
Neg	5126/5457 (93.93)	93.27–94.55	15.46 (13.75–17.39)
**S1-Xpert MTB-RIF**			
Pos	341/6003 (5.68)	5.11–6.3	0.060 (0.05–0.07)
Neg	5662/6003 (94.32)	9.37–94.89	16.58 (14.78–18.60)
S1-Xpert +/S1+	331/396 (83.59)	79.56–87.1	14.71 (12.28–17.66)
S1-Xpert+/S1-	10/341 (2.93)	1.42–5.33	+infinity (3.46- +infinity)
**S2-Xpert MTB-RIF**			
Pos	294/5457 (5.39)	4.8–6.02	0.057 (0.050–0.06)
Neg	5160/5457 (94.56)	93.92–95.14	17.52 (15.49–19.82)
S2-Xpert +/S2+	278/331 (83.99)	79.58–87.7	15.59 (12.78–18.94)
S2-Xpert+/S2-	16/294 (5.44)	3.14–8.69	+infinity (4.38- +infinity)

### Prevalence of Xpert MTB-RIF across smear classification of TB infection

**Fig. 2:**
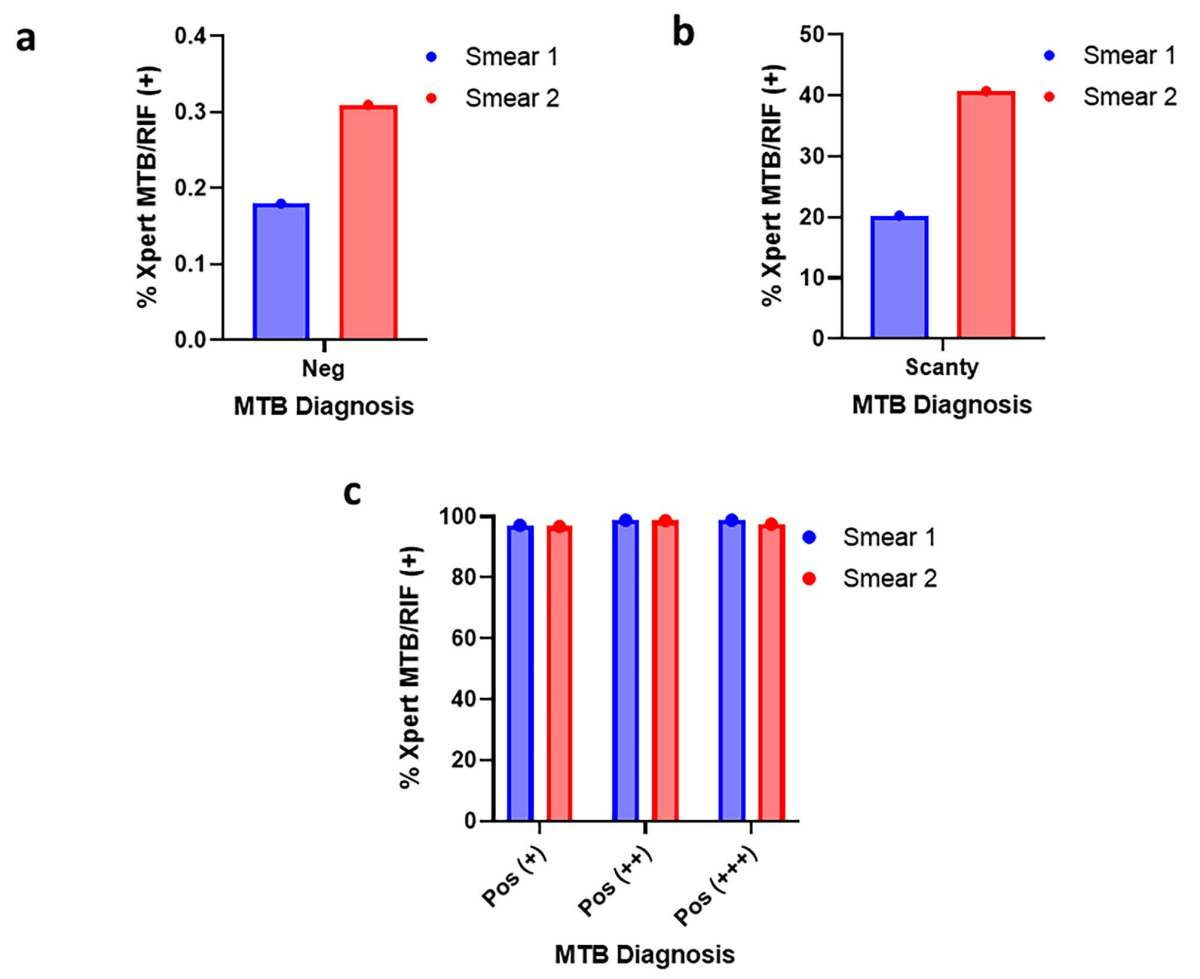
Percentage of GeneXpert MTB/RIF positive cases across smear-classified AFB Microscopic diagnosis. **a**. The overall GeneXpert TB prevalence among smear negative. **b**. The overall GeneXpert TB prevalence among scant smear. **c**. The overall GeneXpert TB prevalence among smear positive cases. MTB- Mycobacteria tuberculosis. RIF-Rifampicin. Negative-no AFB seen. 1 to 9 AFB per 100 fields was considered rare (scanty). 10–99 AFB per 100 fields was considered moderate (positive +). 1–10 AFB per field (check 50 fields) (positive ++). greater than 100 AFB per 100 fields was considered many (positive +++)

The TB smears classification as no infection (negative) 93.4% (5607/6003), scanty 1.2% (74/6003), Positive (+) 2.4% (143/6003), positive (++) 1.5% (92/6003), and positive (+++) 1.4 (87/6003) for the first sputum. A similar pattern of diagnosis was observed for the second sputum. Interestingly, 2.9% (10/341) and 5.4% (16/331) of the Xpert MTB-RIF positive diagnosis were smear-negative for the first and second sputum. There were more positives in the Xpert MTB-RIF diagnosis for the negative and scanty smears in the second TB sputum compared to the first sputum. The scanty samples had very high false positive results of 78.38% (58/74) for smear 1 and 57.89% (44/76) for smear 2. However, the positive samples had low false positive results ranging from 1.09% (1/92) in positive (++) of smear 1 to 2.8% (4/143) in positive (+) of smear 1. Also, false negative smears were 0.18% (10/5607) for smear 1 and 0.31% (16/5126) for smear 2 (Table 2). The Xpert MTB-RIF prevalence for negative smears was 0.18% (10/5607) and 0.31% (16/5126) (Fig. 2 a), scanty smears were 20.27% (15/74) & 40.79% (31/76) (Fig. 2 b), positive (+) were 97.2% (139/143) & 96.77% (90/93), positive (++) were 98.91% (91/92) & 98.73% (78/79) and positive (+++) were 98.85% (86/87) and 97.56% (80/82) for first and second sputum respectively (Fig. 2 c).

**Table 2:** Prevalence of Mycobacteria Tuberculosis diagnosed with smear TB Microscopy and GeneXpert MTB-RIF

		Classification, *n*/*N* (%)			
MTB Test	Neg	Scanty	Pos (+)	Pos (++)	Pos (+++)
Microscopy					
Smear 1	5607/6003 (93.4)	74/6003 (1.2)	143/6003 (2.4)	92/6003 (1.5)	87/6003 (1.4)
Smear 2	5126/5456 (94.0)	76/5456 (1.4)	93/5456 (1.7)	79/5456 (1.4)	82/5456 (1.5)
Xpert MTB test					
Sm1_Xpert MTB-RIF (+)	10/341 (2.9)	16/341 (4.7)	139/341 (40.8)	91/341 (26.7)	86/341 (25.2)
Sm2_Xpert MTB-RIF (+)	16/297 (5.4)	32/297 (10.8)	91/297 (30.6)	78/297 (26.3)	80/297 (26.9)
False positive Smear					
Smear 1	-	58/74 (78.38)	4/143 (2.8)	1/92 (1.09)	1/87 (1.15)
Smear 2	-	44/76 (57.89)	2/93 (2.15)	1/79 (1.27)	2/86 (2.33)
False negative Smear					
Smear 1	10/5607 (0.18)	-	-	-	-
Smear 2	16/5126 (0.31)	-	-	-	-

### TB prevalence among gender and age categories

**Fig. 3:**
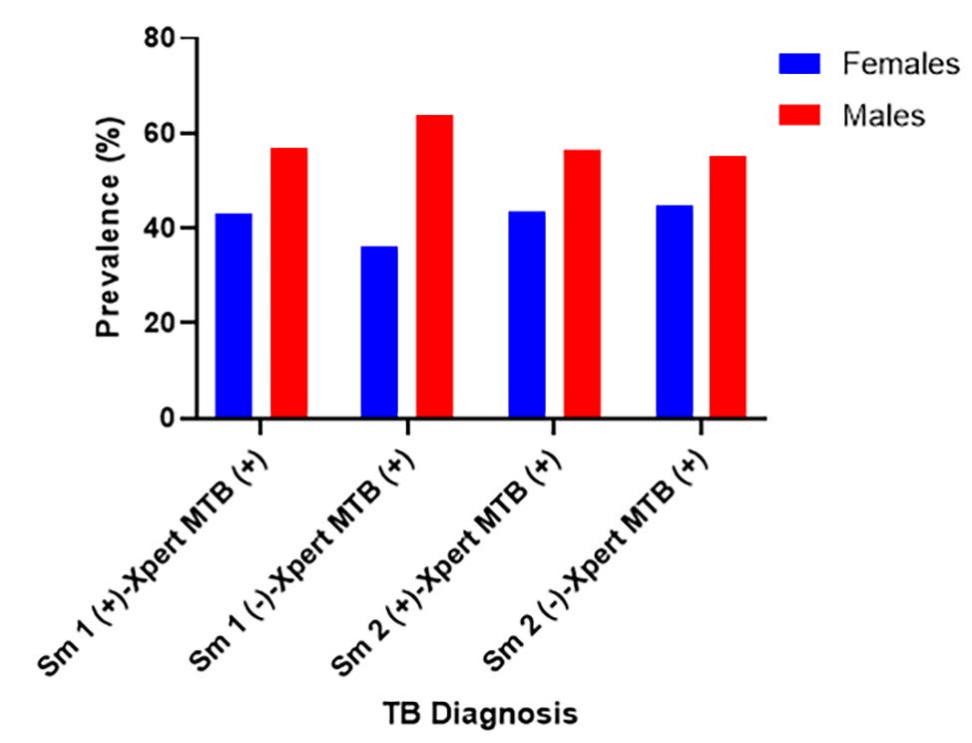
Prevalence of Mycobacteria tuberculosis (MTB) infections by GeneXpert MTB/RIF among males and females with smear-positive and smear-negative cases

**Fig. 4:**
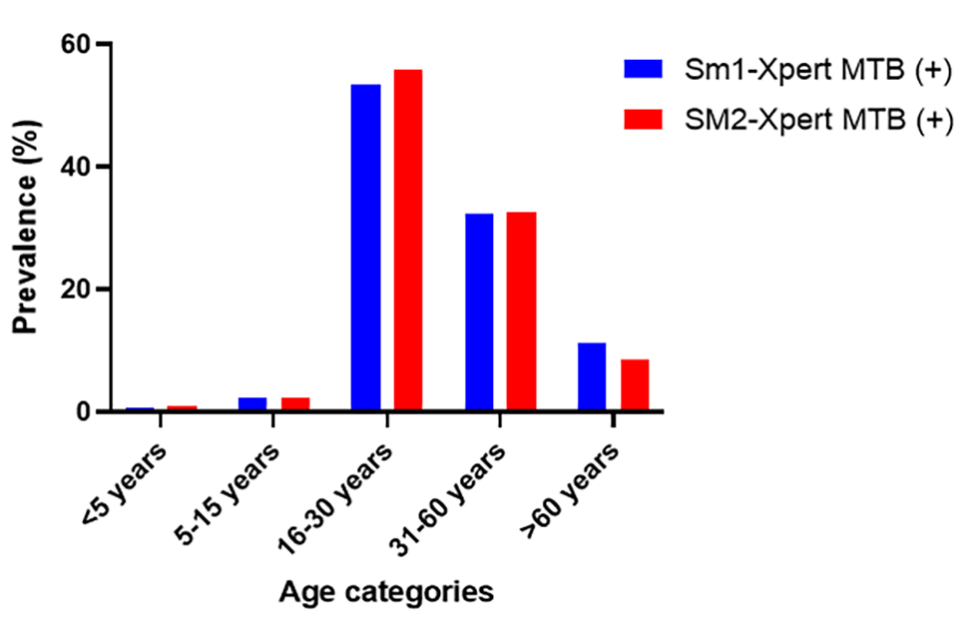
Prevalence of Mycobacteria tuberculosis (MTB) infections by GeneXpert MTB/RIF across age categories

The prevalence of Xpert MTB-RIF across smear positive showed that males had 56.87% (178/313) and 56.15% (137/244) and females had 43.13% (135/313) and 43.85% (107/244) for the first and second sputum (Fig. 3). The Xpert MTB-RIF diagnosis showed that TB is more prevalent among age category 16–30 years (53.2% (174/327) and 55.8% (134/240)) followed by 31–60 years (32.7% (107/327) and 32.5% (78/240)) and > 60 years (11.3% (37/327) and 8.3% (20/240)) in the first and second sputum respectively. The age category < 5 years were group least infected with TB (Fig. 4).

## Discussion

A prompt diagnosis of pulmonary TB plays a significant role in TB disease management, especially in high-endemic TB countries [36]. The impact of accurate and timely TB diagnoses on treatment outcomes and prevention of TB transmission cannot be over-emphasized. Microscopic diagnosis of low TB infections with acid-fast bacilli smear is a challenge for TB management as submicroscopic TB diagnosis contributes to an estimated 12.6% of TB transmission [37]. The GeneXpert MTB-RIF has superior sensitivity and specificity and a rapid turnaround time for diagnosing TB infection [38–40].

The study compared the results of routine diagnoses of TB by microscopy and Xpert MTB from 2016 to 2020 at the Cape Coast Teaching Hospital (CCTH).

The Xpert MTB-RIF diagnosis showed that 2.9% and 5.4% of smears negative TB samples for the first and second sputum harbors *M. tuberculosis*. Similarly, the false positive TB diagnoses with scanty diagnoses have up to 78.38% diagnosed as missed by the Xpert MTB-RIF. The suboptimal microscopic diagnosis of TB smear has explicit or implicit consequences on the National TB Control Program (NTCP) and elimination strategies. The missed diagnosis of pulmonary TB prolongs morbidity and mortality in tuberculosis [41, 42]. Since Microscopy is the mainstay of TB diagnosis in Ghana, an estimated 4.15% missed TB smear diagnosis is evidence of the challenges associated with mapping TB infections, diagnosis, treatment, improving quality of care for patients, and preventing TB transmissions [41, 43, 44].

A previous study reported 2.6% smear-negative but culture positive in Nigeria; the authors recommended TB culture as a confirmatory diagnostic test [45]. The challenge with TB culture is the turnaround time for the results, poor primary healthcare infrastructure, stable electricity, and the skills of laboratory personnel [19, 46]. Also, the GeneXpert MTB/RIF test has reported 16.4% increase in TB detection among cases that were negative in ZN smear microscopy [47, 48]. The study shows that the GeneXpert MTB/RIF test efficiently detects missed ZN TB smear microscopy, similar to the previous studies conducted at Pakistan Institute of Medical Sciences which showed that GeneXpert could detect 15.3% (50/326) MTB DNA among the patients whiles ZN smear microscopy could only detect 9.2% (30/326) of AFB among the same patients [49]. The AFB smear microscopy has a higher detection threshold and lower sensitivity than culture or GeneXpert [50, 51]. Also, microscopic has a high limit of TB detection and can detect TB cells while GeneXpert has low limit of detection and detect the TB DNA. Thus, increasing the sensitivity and specificity of TB diagnosis by GeneXpert when compared with TB smear by microscopic diagnosis [28].

The GeneXpert assay identified 10 and 16 positive MTB from smear 1 and smear 2 samples which were microscopic negative. The result highlights the higher sensitivity of the GeneXpert assay compared to traditional smear microscopy for detecting MTB [52]. The smear microscopy relies on visualizing bacteria under a microscope, which may miss cases with low bacterial load or non-visible bacilli [53]. In contrast, the GeneXpert can detect MTB DNA even at low concentrations, offering higher sensitivity and the ability to detect cases missed by smear microscopy [54–56]. This makes GeneXpert crucial for accurate diagnosis, especially in cases where traditional microscopic methods yield negative results but clinical suspicion remains high.

However, due to the high sensitivity of the GeneXpert assay, it can detect *Mycobacterium tuberculosis* (MTB) DNA without distinguishing between live and dead bacteria [53, 57]. Since the GeneXpert assay detects dead bacteria with intact DNA, a positive result does not distinguish between viable and non-viable bacteria [58]. The detection of the dead MTB DNA in a sample could still be clinically relevant, as it may indicate a previous or ongoing infection, even if the bacteria are no longer actively replicating [59, 60]. However, in making treatment decisions, it is important to distinguish between live and dead bacteria to avoid misuse of drugs and induction of resistant MTB bacteria.

The GeneXpert MTB is estimated to have a detection limit of 136 bacilli/ml of the specimen and provides detection and assessment of the TB resistance to rifampicin, a first-line anti-TB drug [61, 62]. The GeneXpert MTB/RIF provides rapid detection of rifampicin resistance and could prevent transmission from potent smear-negative pulmonary TB [63, 64].

The high male-to-female predominance in TB infections is well known [65, 66]. In low and middle-income countries like Ghana, the high TB prevalence among men is attributed to poor health-seeking behaviour and accessing TB care [67–70]. Men are less likely to have timely TB diagnosis since male TB patients often delay care-seeking longer than female TB patients [70]. The study showed a high prevalence of TB infections among males compared to females in both smear-positive-Xpert positive and smear-negative-Xpert positive cases. The findings are in agreement with the previous studies [66, 67, 70]. Thus, men have a relatively high risk of TB, and disadvantages in accessing TB diagnoses and treatments suggest higher undiagnosed cases of TB in males [71, 72]. Other factors attributed to a high prevalence of TB in men include males being sole breadwinners, lack of disease awareness, working in unorganized sectors, and higher probability of default treatment [71, 72].

The study showed high TB prevalence among the 16–30 years and 31–60 years. The findings suggest that young and older people have a higher risk of TB disease than those below 16 years in this study. Similar studies in Argentina and India have reported slightly higher risks in young adults [73, 74]. In another study, there were significantly lower risks among people under 15 years, and the risks were higher in all groups over 15 years old [75, 76]. Air pollution, smoking, other determinants, continuous TB transmission within the community, staying in cluster environments such as schools, frequent social activities, or disease comorbidities are associated with increased risk exposure to TB disease among specific age groups [76–80].

In conclusion, the study highlights the higher sensitivity of the GeneXpert assay compared to traditional smear microscopy for detecting MTB. The GeneXpert assay identified 10 and 16 positive MTB from smear 1 and smear 2 samples which were microscopic negative. Males are more at risk of TB infections than females. Also, the ages 16–30 years and 31–60 years are more prone to TB infections than the age below 15 years. The sex and age-specific results underscore the importance of understanding demographic characteristics to effectively target high-risk TB populations. Tailored interventions that consider these demographic factors are critical for enhancing TB prevention, early detection, and treatment efforts, ultimately reducing the burden of the disease in vulnerable populations. This demographic insight supports the development of more effective TB control strategies that align with the epidemiological realities of different regions and communities.

## Data Availability

All the data are available in the manuscript and supplementary data.
